# Comparison of Circular and Parallel-Plated Membrane Lungs for Extracorporeal Carbon Dioxide Elimination

**DOI:** 10.3390/membranes11060398

**Published:** 2021-05-27

**Authors:** Leonie S. Schwärzel, Anna M. Jungmann, Nicole Schmoll, Stefan Caspari, Frederik Seiler, Ralf M. Muellenbach, Moritz Bewarder, Quoc Thai Dinh, Robert Bals, Philipp M. Lepper, Albert J. Omlor

**Affiliations:** 1 Department of Internal Medicine V–Pneumology, Allergology and Intensive Care Medicine, University Hospital of Saarland, 66424 Homburg, Germany; leonie.schwaerzel@gmx.de (L.S.S.); a.jungmann@t-online.de (A.M.J.); ni-schmi@gmx.de (N.S.); stefancaspari@gmx.de (S.C.); frederik.seiler@uks.eu (F.S.); thai.dinh@uks.eu (Q.T.D.); robert.bals@uks.eu (R.B.); albert.omlor@uks.eu (A.J.O.); 2 Department of Anaesthesiology and Critical Care, Campus Kassel of the University of Southampton, 34125 Kassel, Germany; ralf.muellenbach@klinikum-kassel.de; 3 Department of Internal Medicine I, University Hospital of Saarland, 66424 Homburg, Germany; moritz.bewarder@uks.eu

**Keywords:** ECCO_2_R, COPD, ARDS

## Abstract

Extracorporeal carbon dioxide removal (ECCO_2_R) is an important technique to treat critical lung diseases such as exacerbated chronic obstructive pulmonary disease (COPD) and mild or moderate acute respiratory distress syndrome (ARDS). This study applies our previously presented ECCO_2_R mock circuit to compare the CO_2_ removal capacity of circular versus parallel-plated membrane lungs at different sweep gas flow rates (0.5, 2, 4, 6 L/min) and blood flow rates (0.3 L/min, 0.9 L/min). For both designs, two low-flow polypropylene membrane lungs (Medos Hilte 1000, Quadrox-i Neonatal) and two mid-flow polymethylpentene membrane lungs (Novalung Minilung, Quadrox-iD Pediatric) were compared. While the parallel-plated Quadrox-iD Pediatric achieved the overall highest CO_2_ removal rates under medium and high sweep gas flow rates, the two circular membrane lungs performed relatively better at the lowest gas flow rate of 0.5 L/min. The low-flow Hilite 1000, although overall better than the Quadrox i-Neonatal, had the most significant advantage at a gas flow of 0.5 L/min. Moreover, the circular Minilung, despite being significantly less efficient than the Quadrox-iD Pediatric at medium and high sweep gas flow rates, did not show a significantly worse CO_2_ removal rate at a gas flow of 0.5 L/min but rather a slight advantage. We suggest that circular membrane lungs have an advantage at low sweep gas flow rates due to reduced shunting as a result of their fiber orientation. Efficiency for such low gas flow scenarios might be relevant for possible future portable ECCO_2_R devices.

## 1. Introduction

Extracorporeal lung support is a promising tool to treat critical lung diseases such as exacerbated COPD, or mild or moderate ARDS, where gas exchange is restricted [1,2]. Two major methods: Extra corporeal membrane oxygenation (ECMO), used for oxygenation and CO_2_ removal of patients in hypoxic and hypercapnic respiratory failure and extracorporeal carbon dioxide removal (ECCO_2_R) only applied in hypercapnic respiratory failure to remove surplus carbon dioxide [3].

In lung failure, the main goal of applying those extracorporeal therapies is, to ensure a sufficient gas exchange and lung protective ventilation to prevent the lung from further damage by invasive ventilation. The Berlin criteria for ARDS state, that the therapy of ARDS requires low tidal volume ventilation with 6 mL/kg body weight and a plateau pressure of 30 mmHg or less [4,5]. Ultra-lung-protective ventilation that uses tidal volumes <6 mL/kg body weight and a plateau pressure of <30 mmHG, in combination with the application of ECCO_2_R, has been proposed by several studies to further improve the outcome [6,7]. The main advantage of the application of ECCO_2_R is the prevention of hypercapnia and respiratory acidosis induced by the reduced ventilation [2,3,6]. Carbon dioxide removal is mainly determined by the sweep gas flow rate and independent from high blood flow rates [3,8,9]. Therefore, in comparison to ECMO, smaller cannulas and membrane lungs with a lower surface area can be used, reducing the risk of bleeding and infections [3,9,10,11].

In general, for ECCO_2_R, there are mainly two predominant types of membrane lungs (MLs): Parallel-plated and circular MLs both promise a sufficient CO_2_ removal at low blood flow rates. In this work, we use an already established mock circuit to compare the two types of MLs and their efficiency on CO_2_ removal at low and ultralow blood flow rates [12].

## 2. Materials and Methods

### 2.1. Standard Protocol

In this work, we used our previously presented ECCO_2_R mock circuit to compare the CO_2_ removal capacity of circular and parallel-plated membrane lungs. The setup consisted of two circuits, the primary and the test circuit.

The primary circuit simulated the human vena cava and was responsible for creating a venous environment by CO_2_ enrichment and O_2_ depletion. In the test circuit, which simulated the ECCO_2_R device connected to the vena cava, the CO_2_ removal rate of four different membrane lungs was tested. The whole setup was filled with fresh porcine blood as test fluid. The primary circuit consisted of a Getinge Quadrox PLS (Permanent Life Support) membrane lung built into a loop circuit with a Rotaflow centrifugal pump and 3/8″polyvinyl chloride (PVC) tubings. To create a venous environment, N_2_ and CO_2_ were applied as sweep gases to the Quadrox PLS at rates of 7.5 L/min N_2_ and 0.55 L/min CO_2_ to create a CO_2_ partial pressure in blood p_CO_2_(blood)_ between 43 mmHg ± 2 mmHg and a venous oxygen saturation of 65 ± 5% (data not shown). A blood flow of 5 L/min was chosen to operate the primary circuit according to the human cardiac output. The system was tempered to 37 °C with a Maquet HU 35 via the Quadrox PLS membrane lung.

**Figure 1:**
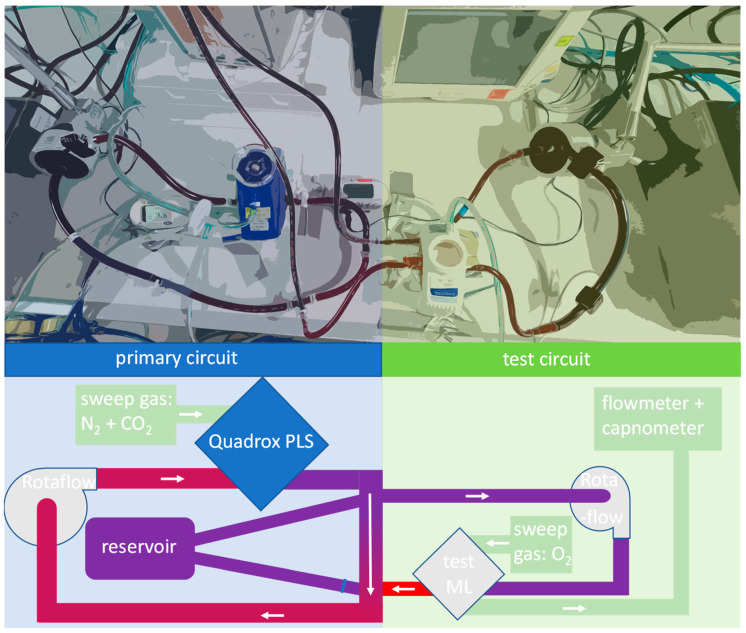
Photographic and schematic view of our mock setup. The mock setup was filled with heparinized fresh porcine blood. The primary circuit simulated the human vena cava and generated venous blood by applying a sweep gas of N_2_ and CO_2_ to a Quadrox PLS membrane lung. The test circuit simulated the actual ECCO_2_R setup connected to the vena cava. In this experiment, comparable circular and parallel-plated membrane lungs were sequentially built into the test setup as test membrane lung (ML). The CO_2_ removal rate, which was calculated from gas flow rate and CO_2_ content at the outlet of the membrane lungs, was compared between circular and parallel-plated membrane lungs.

Via Luer Lock connectors, the test circuit with the membrane lung (ML) to be tested was connected according to Figure 1. The blood flow through these Luer Lock connectors was called cannula flow in this work. Pure oxygen was applied as sweep gas to the test membrane lung. At the sweep gas outlet of the test ML, a mass flow sensor (TSI41403) and a side-flow capnometer (Philips Intellivue) were installed.

CO_2_ removal rates Q_CO_2__ (N = 6 for each group) were calculated from the sweep gas flow rate Q_sweep_ at the sweep outlet, the partial pressure of CO_2_ in the sweep gas p_CO_2_(gas)_ and the atmospheric pressure p_atm_ according to the following formula:

Q_CO_2__ = Q_sweep_ ∗ p_CO_2_(gas)_/p_atm_ (1)

### 2.2. Monitoring of the Test Fluid

The setup was filled with fresh porcine blood, added with 10.000 IE Heparin to avoid clotting and 1g Meropenem to avoid bacterial growth during the measurement. Blood gas analysis was performed to monitor the quality of the blood. Throughout the whole measurement series blood gas analysis confirmed that hemoglobin was constantly at 15 g/dl. PH was in range of 7.40 ± 0.20, bicarbonate in the rage from 23–26 mmol/L, venous saturation of oxygen in the range of 65% ± 5 %. Lactate values of up to 10 mmol/L at the beginning of the measurement were tolerated.

### 2.3. Comparison of Membrane Lungs

Of the four test membrane lungs, two were parallel-plated MLs (Maquet Quadrox-i Neonatal, Maquet Quadrox-iD Pediatric) and two were circular (Medos Hilite 1000, Novalung MiniLung).

**Figure 2:**
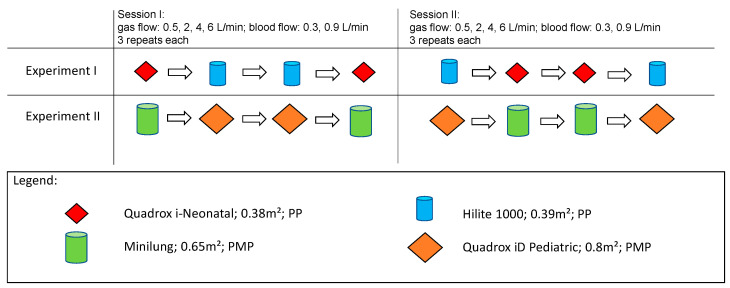
Measurement order of the MLs in session I and II: In each session, two experiments were conducted. In experiment I, the two low-flow membrane lungs were compared: The parallel-plated Quadrox-i Neonatal and the circular Hilite 1000. In experiment II, the two mid-flow membrane lungs were compared: The parallel-plated Quadrox-iD Pediatric and the circular Minilung. Each session had four symmetrical measuring slots to prevent an advantage of one ML due to fresher blood. Therefore, the membrane was either measured in slot 1 and slot 4 or in slot 2 and slot 3. In session II, the order was swapped.

In general, two sessions, measuring all 4 MLs, were performed. Each session consisted of 2 experiments (Figure 2). For each ML, blood flow (BF) rates were set at 0.3 L/min and 0.9 L/min. Moreover, for each blood flow rate, the sweep gas flow (Q_sweep_) was set at 6, 4, 2 and 0.5 L/min. In each session, a total of 6 repeated measurements were performed for each BF and Q_sweep_ configuration. For each session, new MLs were chosen, blood samples were changed for each experiment. In session I, experiment I, the low-flow MLs, the Quadrox i-Neonatal with a surface area (SA) of 0.38 m² and the Hilite 1000 oxygenator with a surface area of 0.39 m², both consisting of polypropylene (PP) fibers, were compared. In session I, experiment II, the mid-flow MLs, Quadrox-iD Pediatric with a SA of 0.8 m² and the Novalung Minilung ML with a SA of 0.65 m², both consisting of polymethylpentene (PMP) fibers, were compared. In session II, performed on a separate day, the two experiments were repeated with new MLs. Each session had four measuring slots per experiment, as each ML was measured twice. The membrane lung that was tested in the first slot, would also be tested in the last slot. In session II, the measurements were repeated with new blood and MLs. The order was also modified, so that a membrane design, that had been tested in the first and last slot in session I, was tested in the second and third slot.

### 2.4. Statistics

Statistical analysis was performed with GraphPad Prism 5.02 (GraphPad Software, Inc., La Jolla, CA). Data were presented as means ± SEM. The Kolmogorov–Smirnov test was used to evaluate that all groups showed Gaussian distribution. Differences between groups were tested with the unpaired *t*-test. *P*-values <0.05 (*) and <0.01 (**) were considered significant. *P*-values <0.001 (***) were considered highly significant.

## 3. Results

**Figure 3:**
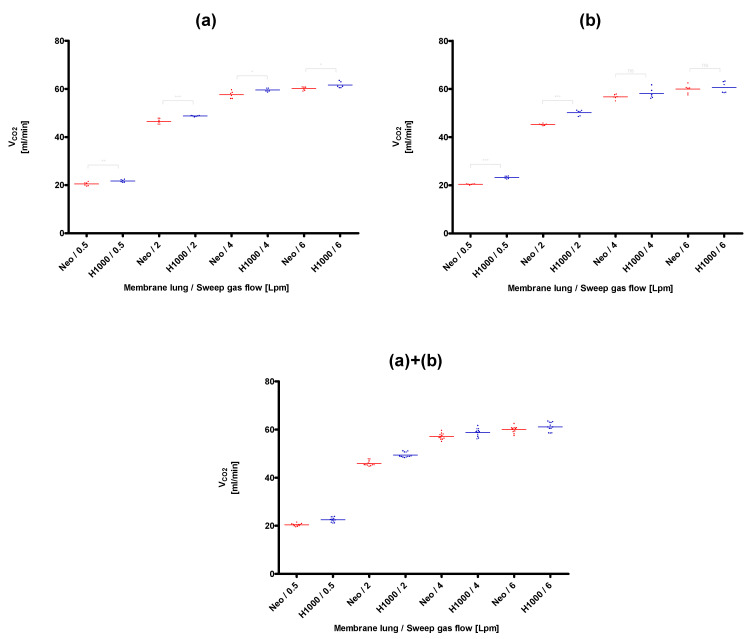
Comparison of the CO_2_ removal performance of the Quadrox-i Neonatal (Neo) and the Hilite 1000 (H1000) at a blood flow of 0.3 L/min. (**a**): Session I, (**b**): Session II, (**a**)+(**b**): Combination of session I and II. Both sessions were performed on separate days with new blood samples and MLs.

**Figure 4:**
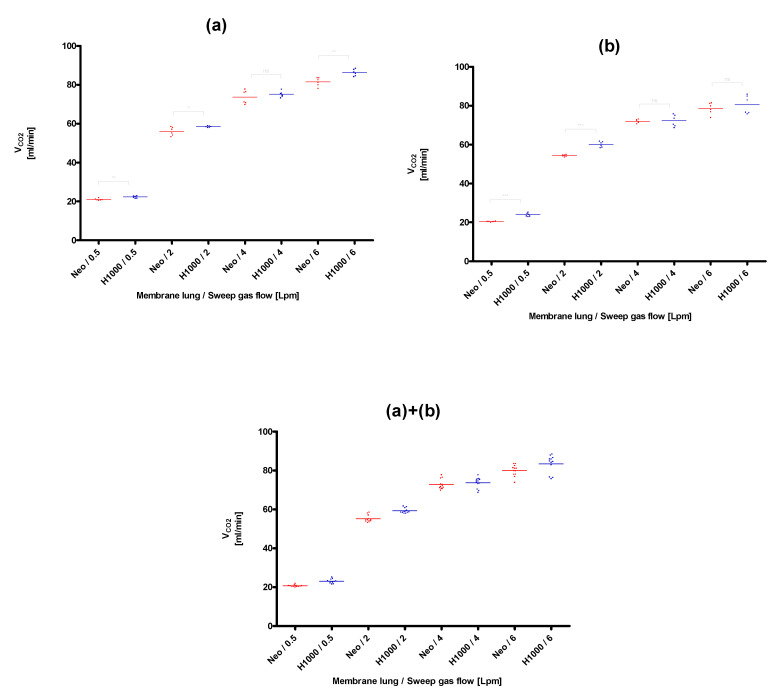
Comparison of the CO_2_ removal performance of the Quadrox-i Neonatal (Neo) and the Hilite 1000 (H1000) at a blood flow of 0.9 L/min. (**a**): Session I, (**b**): Session II, (**a**)+(**b**): Combination of session I and II. Both sessions were performed on separate days with new blood samples and MLs.

**Figure 5:**
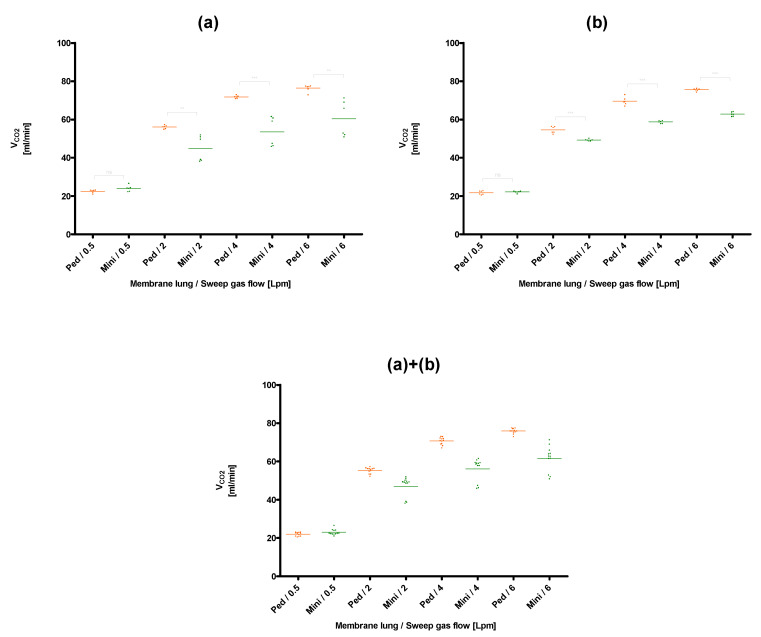
Comparison of the CO_2_ removal performance of the Quadrox-iD Pediatric (Ped) and the Minilung (Mini) at a blood flow of 0.3 L/min. (**a**): Session I, (**b**): Session II, (**a**)+(**b**): Combination of session I and II. Both sessions were performed on separate days with new blood samples and MLs.

**Figure 6:**
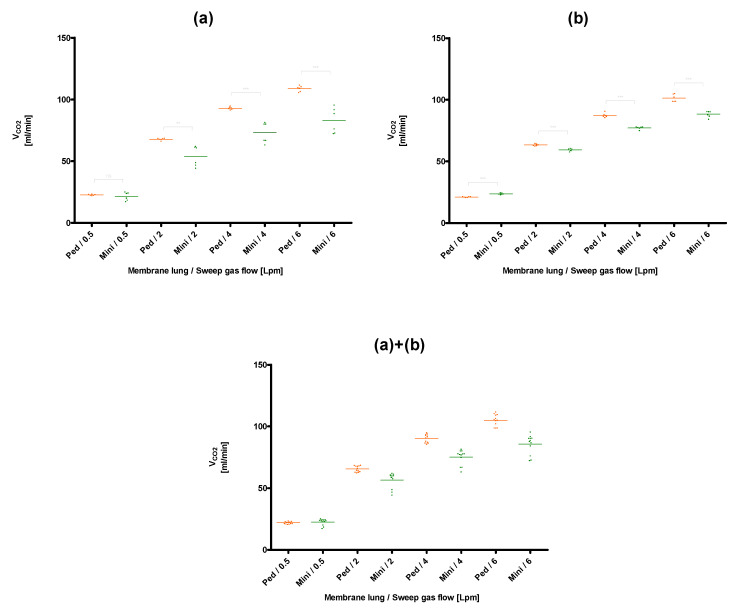
Comparison of the CO_2_ removal performance of the Quadrox-iD Pediatric (Ped) and the Minilung (Mini) at a blood flow of 0.9 L/min. (**a**): Session I, (**b**): Session II, (**a**)+(**b**): Combination of session I and II. Both sessions were performed on separate days with new blood samples and MLs.

The lowest CO_2_ removal rate (20.4 ± 0.2 mL/min) was found for the membrane lung with the smallest surface area, the Quadrox-i Neonatal, at the lowest blood (0.3 L/min) and sweep gas flow rates (0.5 L/min), that were tested (Figure 3). The best CO_2_ removal performance (105.0 ± 1.3 mL/min) was observed for the membrane lung with the highest SA, the Quadrox-iD Pediatric at the highest blood flow (0.9 L/min) and sweep gas flow (6 L/min). The Q_CO_2__ increased in a nonlinear rate both with an increased sweep gas (Figure 3, Figure 4, Figure 5 and Figure 6) for both the circular and parallel-plated membrane lungs. For the higher blood flow of 0.9 L/min, increased CO_2_ removal rates were observed. However, the increase was not proportional (Figure 4 and Figure 6).

In the comparison of the two low-flow PP fiber membrane lungs, the circular Hilite 1000 demonstrated an overall better performance than the parallel-plated Quadrox-i Neonatal for all sweep gas flow rates and for both evaluated blood flow rates of 0.3 L/min (Figure 3) and 0.9 L/min (Figure 4). The most significant advantages in CO_2_ removal of the Hilite 1000 over the Quadrox-i Neonatal were observed at the low sweep gas rate of 0.5 L/min. At higher sweep gas flow rates (4, 6 L/min), the differences of the two low-flow MLs were not always significantly different.

In the comparison of the mid-flow PMP fiber membrane lungs, the parallel-plated Quadrox-iD Pediatric showed significantly higher CO_2_ removal rates than the respective circular Minilung for both blood flow rates and the sweep gas flow rates of 2, 4, and 6 L/min (Figure 5 and Figure 6). For the Q_sweep_ of 0.5 L/min, the advantage in the CO_2_ removal of the Quadrox-iD Pediatric cannot be observed anymore. Rather, a tendency of a benefit of the Minilung with a blood flow rate of 0.9 L/min can be shown in the second session (Figure 6).

**Figure 7:**
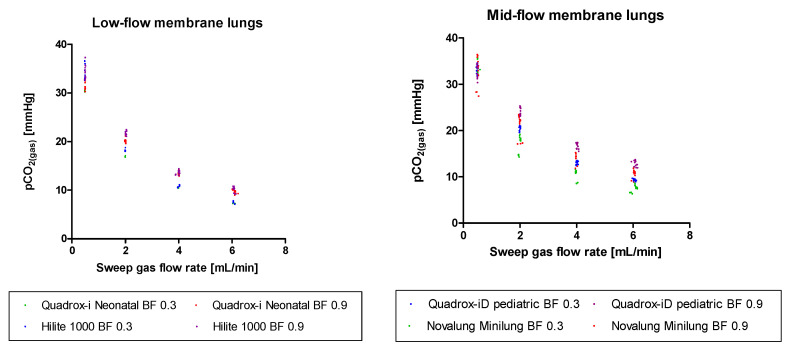
Comparison of the p_CO_2_(gas)_ of the parallel-plated and circular ML at Q_sweep_ configurations of 0.5, 2, 4, 6 L/min and a BF of 0.3, 0.9 L/min. A: Comparison of the p_CO_2_(gas)_ of the Quadrox-i Neonatal and the Hilite 1000. B: Comparison of the p_CO_2_(gas)_ of the Quadrox-iD Pediatric and the Novalung Minilung.

Moreover, the p_CO_2_(gas)_ vs the sweep gas flow rate was displayed in Figure 7 for each ML. It showed, that for all MLs, at low sweep gas flow rates of 0.5 L/min, p_CO_2_(gas)_ approximates the p_CO_2_(blood)_, which was 43 ± 2 mmHg in our measurements. The greater Q_sweep_, the lower p_CO_2_(gas)_ is measured at the sweep gas outlet of the MLs.

## 4. Discussion

In this work, the efficiency in CO_2_ removal of parallel-plated oxygenators and circular oxygenators was evaluated with our already established mock circuit. The CO_2_ removal rate of the Quadrox-iD Pediatric, the standard oxygenator for ECCO_2_R in our unit, was in range with earlier results [12,13].

Few comparisons of different MLs can be found in the literature [10,14,15,16,17]. However, most of these works only focus on the efficiency of the compared MLs. Surface area, fiber structure and construction of the oxygenator were not completely respected [14,15,16]. Rambaud et al. compared a parallel-plated oxygenator to a circular oxygenator on the CO_2_ removal capacity in newborn patients and demonstrated a benefit of the circular oxygenator [17]. However, the surface area of the compared MLs was different, as the study compared the mid-flow membrane lung Quadrox-iD Pediatric (0.8 m^2^) to the low-flow membrane lung Medos Hilite 800LT (0.32 m^2^) [17]. Although the CO_2_ removal rates were normalized to the surface area, it remains debatable whether there is a linear relation over such a large range for the SA. In fact, Karagiannidis et al. showed a strong, but not linear, impact of the membrane SA on CO_2_ removal in his study with pigs [10]. However, this study used only parallel-plated MLs, and the effect of an increased membrane area varied for different blood flow rates. Hence, to make a reasonable comparison of two MLs, surface area, fiber structure, blood flow and sweep gas flow need to be equal. 

Therefore, in our study we did not use normalization but rather tried to minimize the difference in membrane area of the compared membrane lungs.

Hence, the Novalung Minilung, a rebranded Medos Hilite 2400LT, with a surface area of 0.65 m^2^ rather than a Medos Hilite 800LT was used as the circular comparison to the Quadrox-iD Pediatric, with 0.80 m^2^ SA. Although the match of the surface area is not perfect, it was the closest we could get for parallel-plated and circular membrane lungs in the mid-flow range.

For the low-flow MLs, we used polypropylene MLs because, according to our knowledge, no commercially available low-flow parallel plated polymethylpentene MLs are produced. In this category, the two MLs, Quadrox-i-Neonatal (0.38 m^2^) and Medos Hilite 1000 (0.39 m^2^) were closer in surface area than their mid-flow counterparts.

The overall higher CO_2_ removal rates of the circular oxygenator Hilite 1000 in the low-flow contest and the Quadrox-iD Pediatric in the mid-flow contest were unsurprising, as both membranes surpassed their counterpart in terms of SA. More interesting was, however, how the relative performance differences shifted, when different sweep gas flow rates were used. For the lowest tested gas flow of 0.5 L/min, the parallel-plated Quadrox-iD Pediatric lost its lead, while the circular Hilite 1000 even increased its relative advantage.

All in all, we see a slight advantage of circular MLs for low sweep gas flow rates. We contribute this to the principal design of the membrane lungs. While the parallel-plated MLs have a blood flow perpendicular to the gas fiber orientation, the circular MLs have a blood flow that is almost antiparallel to the gas fibers. When the sweep gas travels along the gas fibers, it accumulates CO_2_. At low sweep gas flow rates, the CO_2_ partial pressure at the distant end of the gas fibers is close to the CO_2_ partial pressure in blood (Figure 7). As a result, this part of the membrane lung has an inefficient CO_2_ removal due to a small diffusion gradient.

**Figure 8:**
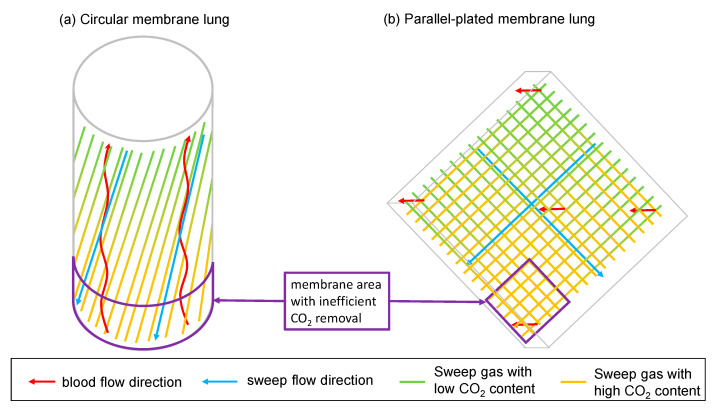
Fiber arrangement of parallel-plated and the circular MLs. (**a**): For the circular ML, blood flow is almost antiparallel to the gas fibers and the gas flow. At the lower part of the ML, where blood enters, it is in contact with the sweep gas with the highest amount of CO_2_. As diffusion of CO_2_ through the ML depends on the diffusion gradient between gas and blood, which is small in this part of the ML, the CO_2_ removal in this area is inefficient. (**b**): For the parallel-plated MLs, blood flow is perpendicular to the gas fibers. At the lower part of the ML where gas with highest CO_2_ content is in contact with blood, CO_2_ removal is inefficient.

In case of the circular membrane lung, the lower part of the oxygenator (according to the arrangement in Figure 8), where the blood enters, is affected by the adjustment of the CO_2_ partial pressure in gas and in blood. For the parallel-plated membrane lung this effect is more complex, as it has alternating layers of perpendicular orientated gas fibers. Therefore, the upper part of the membrane lung (according to the arrangement in Figure 8) has sweep gas with low CO_2_ content in both fiber orientations. The left and right parts of the membrane lung have sweep gas with low CO_2_ content in one and sweep gas with high CO_2_ content in the other fiber orientation, while the lower part of the membrane lung has sweep gas with high CO_2_ content in both fiber orientations. As a consequence, a blood fraction that passes the parallel-plated membrane lung in the lower part, will shunt to the blood outlet with little to no CO_2_ removal. As shown in Figure 7, for a sweep gas flow of 0.5 L/min, the CO_2_ partial pressure in sweep gas at the sweep gas outlet of the membrane lung gets close enough to the CO_2_ partial pressure in blood for this effect to be relevant. However, in the circular membrane lung, due to the antiparallel arrangement, all blood must pass the membrane area with inefficient CO_2_ removal first, but after that also passes a membrane area with efficient CO_2_ removal. Therefore, in this membrane lung design, no blood can shunt, independent of the sweep gas flow rate.

The most notable limitation of this study is the fact that there are no perfectly matched MLs for the circular and the parallel-plated design. Although we tried to use as similar membrane lungs as possible, the surface area remains a strong unmatched confounder.

Another critical factor for CO_2_ removal is the freshness of the used blood fluid itself. Ideally, for each session, both compared membranes would have to be tested at the same time with the same blood sample. As this is not possible to perform, we designed a symmetrical measuring sequence as shown in Figure 2 that minimizes the possible disadvantage of the ML measured second due to a less fresh or different blood sample. In our setup, for each experiment, each ML is tested two times. To overcome the disadvantage, the ML that is measured in the first slot will be measured in the last slot again. Moreover, in the second session, where the experiment is repeated with new MLs and a new blood sample, the measurement order is changed. That means that the ML that was measured in the first and in the last slot in the first session, was measured in the two middle slots of the second session and vice versa. Because of this arrangement, we have high confidence in the comparability of the measurements.

However, the performance comparison was only tested for new membrane lungs. We did not observe CO_2_ removal rates over longer time frames of several days. The longer a ML runs, the more clotting occurs within the ML, reducing the CO_2_ removal performance. It is possible that these deterioration processes are faster in some ML designs than in others. Therefore, the CO_2_ removal performance over longer time frames must be addressed in future experiments.

All in all, we conclude that circular MLs are more efficient in CO_2_ removal than parallel-plated MLs at low sweep gas flow rates. Such low sweep gas flow rates might not be relevant for everyday clinical practice of patients in acute respiratory distress syndrome and are only observed in the process of weaning a patient from ECMO or ECCO_2_R [18]. However, we think that our results might be relevant in the design of specialized ECMO devices, such as a portable ECCO_2_R, that needs to be extremely efficient with its limited sweep gas supply.

## Data Availability

Data can be provided on request addressed to the corresponding author. All data sharing statements are subject to conformity with German data protection legislation and rules (Datenschutzgrundverordnung—DGSVO).
